# SpineDepth: A Multi-Modal Data Collection Approach for Automatic Labelling and Intraoperative Spinal Shape Reconstruction Based on RGB-D Data

**DOI:** 10.3390/jimaging7090164

**Published:** 2021-08-27

**Authors:** Florentin Liebmann, Dominik Stütz, Daniel Suter, Sascha Jecklin, Jess G. Snedeker, Mazda Farshad, Philipp Fürnstahl, Hooman Esfandiari

**Affiliations:** 1 Research in Orthopedic Computer Science, Balgrist University Hospital, University of Zurich, 8008 Zurich, Switzerland; dstuetz@student.ethz.ch (D.S.); daniel.suter@balgrist.ch (D.S.); sascha.jecklin@balgrist.ch (S.J.); philipp.fuernstahl@balgrist.ch (P.F.); hooman.esfandiari@balgrist.ch (H.E.); 2 Laboratory for Orthopaedic Biomechanics, ETH Zurich, 8093 Zurich, Switzerland; jess.snedeker@balgrist.ch; 3 Computer Vision and Geometry Group, ETH Zurich, 8093 Zurich, Switzerland; 4 Department of Orthopedics, Balgrist University Hospital, University of Zurich, 8008 Zurich, Switzerland; mazda.farshad@balgrist.ch

**Keywords:** data generation, artificial intelligence, RGB-D, surgical navigation, spinal fusion, pedicle screw placement, registration, calibration

## Abstract

Computer aided orthopedic surgery suffers from low clinical adoption, despite increased accuracy and patient safety. This can partly be attributed to cumbersome and often radiation intensive registration methods. Emerging RGB-D sensors combined with artificial intelligence data-driven methods have the potential to streamline these procedures. However, developing such methods requires vast amount of data. To this end, a multi-modal approach that enables acquisition of large clinical data, tailored to pedicle screw placement, using RGB-D sensors and a co-calibrated high-end optical tracking system was developed. The resulting dataset comprises RGB-D recordings of pedicle screw placement along with individually tracked ground truth poses and shapes of spine levels L1–L5 from ten cadaveric specimens. Besides a detailed description of our setup, quantitative and qualitative outcome measures are provided. We found a mean target registration error of 1.5 mm. The median deviation between measured and ground truth bone surface was 2.4 mm. In addition, a surgeon rated the overall alignment based on 10% random samples as 5.8 on a scale from 1 to 6. Generation of labeled RGB-D data for orthopedic interventions with satisfactory accuracy is feasible, and its publication shall promote future development of data-driven artificial intelligence methods for fast and reliable intraoperative registration.

## 1. Introduction

Computer aided orthopedic surgery (CAOS) was introduced approximately three decades ago and it has been referred to as one of the notable advancements in medicine. While CAOS has been shown to result in superior accuracy compared to conventional free-hand surgery in interventions such as spinal fusion [1,2], a world-wide survey in 2013 reported that only 11% of such interventions are performed using CAOS systems [3]. Furthermore, clinical acceptance of such systems in orthopedic procedures has been reported to be incremental [4] with conservative estimates projecting their usage rate to be less than 5% of surgeries performed in North America, Europe and Asia [5]. Factors such as steep learning curves, line-of-sight issues, poor ergonomics and economics of existing CAOS solutions are noted as the main reasons for the low rate of clinical adoption [5,6]. Many CAOS systems rely on registration of the target anatomy to a preoperative plan and real-time tracking of surgical instruments to provide intraoperative surgical navigation. Registration is commonly achieved by either manual identification of mutual points and/or surfaces on the target anatomy and the preoperative volumetric images or by employing image-based 2D–3D or 3D–3D registration techniques where preoperative data is matched to intraoperative ultrasound, fluoroscopy or Cone-Beam Computed Tomography (CBCT) images [6,7]. Such registration methods are technically demanding, error prone and cumbersome due to factors such as patient movement, instability of the attached reference markers, intraoperative radiation exposure and small capture range issues (in case of 2D–3D registration methods [8]).

The emerging consumer-grade, low-cost RGB-D sensors enable new opportunities for improvement of the abovementioned registration procedures by providing direct real-time 3D representation of the exposed anatomy. Several studies have investigated the use of such sensors for registration of intraoperative anatomy, providing depth-based surgical navigation. The authors in [9] utilized a device combining RGB images and active stereoscopy depth sensing for registration of a preoperative femur 3D model in total knee arthroplasty. A deep convolutional neural network was trained to segment intraoperative RGB images and therewith the corresponding depth data, which was used for registration of the preoperative 3D model. An extension of their work additionally included segmentation and registration of the tibia [10]. Registration of an RGB-D sensor (attached to a mobile C-arm gantry) to intraoperative CBCT imaging was achieved in [11,12] by performing the Iterative Closest Point (ICP) technique on a calibration object visible both in the CBCT image and the RGB-D stream. Real-time tracking and augmented reality overlay of glenoid in total shoulder arthroplasty was developed in [13], where the built-in time of flight (ToF) sensor of a HoloLens (Microsoft Corporation, Redmond, WA, USA) was used as the depth data source to register the preoperative 3D model to the anatomy after a coarse manual alignment. However, except for a system using more expensive, proprietary hard- and software [14,15], depth-based surgical navigation could not translate into clinical practice yet because sensor accuracy and robustness are still unsatisfying, and the registration of the depth data to the anatomy is mainly achieved through conventional error-prone means (e.g., surface-based or point-based registration). For infrared-based ToF sensors, material dependent depth measurements have been identified as one major source of error [10,11,13]. More generally, comprehensive studies comparing depth accuracy of sensors using different technologies (structured light, ToF and active/passive stereoscopy) report ambiguous results between 0.6 mm and 3.9 mm error at 600 mm [16,17,18], which can be a realistic distance in a surgical environment.

A potential for improving accuracy and workflow is to leverage new data-driven methods of artificial intelligence that allow for direct reconstruction of 3D shapes or estimation of 3D object poses based on RGB-D data (e.g., [19,20,21,22,23]). Such methods have the potential to alleviate the shortcomings of conventional registration techniques in surgical navigation. However, their implementation in the medical field requires large training datasets of intraoperative RGB-D streams along with corresponding ground truth 3D shapes and poses of the target anatomy. To the best of our knowledge, existing datasets only cover natural or industrial images and objects (e.g., [24,25]); therefore, the objective of the herein presented study was to develop a framework that enables the acquisition of large clinical data (tailored to pedicle screw placement procedure in spinal fusion surgery) for future method development. This is achieved by recording mock up surgeries performed on cadaveric specimens in a realistic OR environment using two RGB-D sensors, while simultaneously tracking the anatomy with a co-registered high-end Optical Tracking System (OTS). Our setup is designed in a way that no component of that system is visible within the surgical site in the RGB-D stream. Our primary contributions are (1) a detailed methodological description of the data collection setup, (2) a large public dataset of RGB-D recordings that include both the ground truth 3D vertebra poses and 3D vertebral shapes and (3) quantitative and qualitative evaluation estimating the accuracy of the setup as well as the collected data.

## 2. Materials and Methods

The following highlights the sequence of material presented in the remainder of this article: a short statement regarding the choice of sensor, an overview of the data acquisition setup, a detailed description of the extrinsic calibration process, the cadaver preparation, the data acquisition, the data post-processing, the outcome measures and the depth correction. The following mathematical notations are used throughout this article: a coordinate frame A is denoted as $F_{A}$. A transformation from $F_{A}$ to $F_{B}$ is denoted as ${}^{B}T_{A}$. Static transformations are depicted as solid lines, whereas time-dependent transformations are denoted as ${}^{B}T_{A}\left(t\right)$ and depicted as dashed lines. A point set *P* expressed in $F_{A}$ is denoted as ${}^{A}P$.

### 2.1. Choice of Sensor

A pre-evaluation of RGB-D sensors was conducted for justifying our sensor selection. Comparisons were based on a working distance of 600 mm, which was assumed to be realistic in a surgical setting. Depth estimation is commonly based on structured light, time of flight (ToF) or active/passive stereoscopy technology. Objective quantification of the depth accuracy of a given RGB-D sensors is a challenging task. A generic method that allowed for the expression of depth error as a function of physical distance between the sensor and a chessboard target was proposed in [16]. An error of 1.1 mm was estimated for Kinect 2 (ToF; Microsoft Corporation, Redmond, WA, USA) and 9.4 mm error was reported for ZED (passive stereoscopy; Stereolabs Inc., San Francisco, CA, USA), respectively. Depth errors of Intel RealSense D415 (active stereoscopy; Intel, Santa Clara, CA, USA) and Stereolabs ZED Mini (passive stereoscopy) were assessed in [17] using 3D printed fixtures equipped with optically tracked reference markers. For the D415, an error of 2.2 mm was found. The error for the ZED Mini was 3.9 mm. A comprehensive study involving D415, ZED Mini, Kinect 2 and Intel RealSense SR300 (structured light) was published in [18] following an approach similar to [16]. The authors suggested that the SR300 yields the most accurate depth measurements, but its structured light technology prevents it from being used in a multi-camera setup. The remaining three sensors showed mean depth errors of 1.0 mm (D415), 1.2 mm (ZED Mini) and 1.6 mm (Kinect 2).

Summarizing the above-mentioned results, the sensors can operate in a similar range of accuracy, but with different application-dependent advantages and disadvantages. In particular, for application of this study we chose the ZED Mini sensor based on the following three factors: (1) it is suited for multi-camera setups (unlike structured light sensors), (2) it is not infrared-based (where measurements can be material-dependent) while being similarly accurate at close range, and (3) the recorded data allows for versatile post-processing and real time applications due to high-resolution stereoscopic RGB images.

### 2.2. Data Acquisition Setup

**Figure 1:**
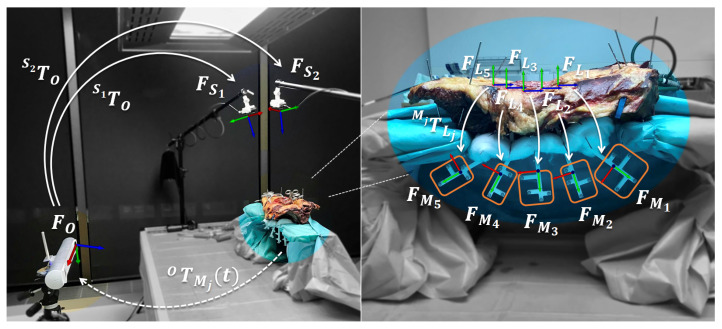
Setup overview with coordinate frames (*F*) and transformations (*T*). Dedicated markers ($F_{M_{1}},F_{M_{2}},\ldots ,F_{M_{5}}$) are attached to spine levels L1–L5 ($F_{L_{1}},F_{L_{2}},\ldots ,F_{L_{5}}$), enabling individual tracking (${}^{O}T_{M_{j}}\left(t\right)$) by the optical tracking system (OTS; $F_{O}$). The surgical site is viewed from the top by two RGB-D sensors ($F_{S_{1}},F_{S_{2}}$). Extrinsic calibrations between OTS and RGB-D sensors (${}^{S_{1}}T_{O},{}^{S_{2}}T_{O}$) can be found intraoperatively using the method described in Section 2.3. The relative marker poses w.r.t. the anatomy (${}^{M_{j}}T_{L_{j}}$) are determined in a postoperative CT (Section 2.6).

Figure 1 provides an overview of the data collection setup. The goal was to capture the surgical site from viewpoints of an RGB-D sensor mounted on a surgeon’s head or an OR light along with ground truth 3D pose and shape data of spinal levels L1–L5. Three modalities were involved: two RGB-D sensors, an OTS (fusionTrack 500, Atracsys LLC, Puidoux, Switzerland) and postoperative CT. The RGB-D sensors were positioned above the surgical site, facing the anatomy ($F_{S_{1}}$ and $F_{S_{2}}$). The OTS was responsible for ground truth tracking of the anatomy and was placed on the left side of the table ($F_{O}$), opposite of the surgeon, who worked from the right side. In order to transform such data to the coordinate frame of either RGB-D sensor (${}^{S_{1}}T_{O}$, ${}^{S_{2}}T_{O}$), an extrinsic calibration process was needed (Section 2.3). Due to possible intervertebral movement during surgery, each vertebra was equipped with a dedicated 3D printed marker ($F_{M_{1}},F_{M_{2}},\ldots ,F_{M_{5}}$) and tracked individually by the OTS (${}^{O}T_{M_{j}}\left(t\right),\;j\in \left\{1,2,\ldots ,5\right\}$). The markers were designed according to the principle of patient-specific instruments (Section 2.4) and protruded from the specimen in anterior direction, facing the OTS. Free movement of the vertebrae and the attached markers was ensured by two custom-made, C-shaped wooden jigs, which elevated the specimen from the table. During data acquisition (Section 2.5), the surgeon placed ten pedicle screws bilaterally on levels L1–L5, captured simultaneously by the two RGB-D sensors and the OTS. Furthermore, recordings for accuracy evaluation and a postoperative CT were conducted. Post-processing of the recordings and the postoperative CT (Section 2.6) yielded all necessary data for generation of labeled RGB-D frames and setup accuracy evaluation. Most importantly, the transformation between anatomy and markers (${}^{M_{j}}T_{L_{j}}$), where $L_{j}$ is the local coordinate frame of a given vertebra level *j*, was determined from the postoperative CT. This completed the transformation chain, which enables the expression of anatomy data in RGB-D coordinate frames:

(1) $${}^{S_{i}}T_{L_{j}}\left(t\right)={}^{S_{i}}T_{O}\;{}^{O}T_{M_{j}}\left(t\right)\;{}^{M_{j}}T_{L_{j}},\;\;i\in \left\{1,2\right\},\;j\in \left\{1,2,\ldots ,5\right\}.$$

Therewith, the quantitative and qualitative outcomes measures (Section 2.7) estimating the accuracy of the setup and the recorded dataset could be calculated.

### 2.3. Extrinsic Calibration

**Figure 2:**
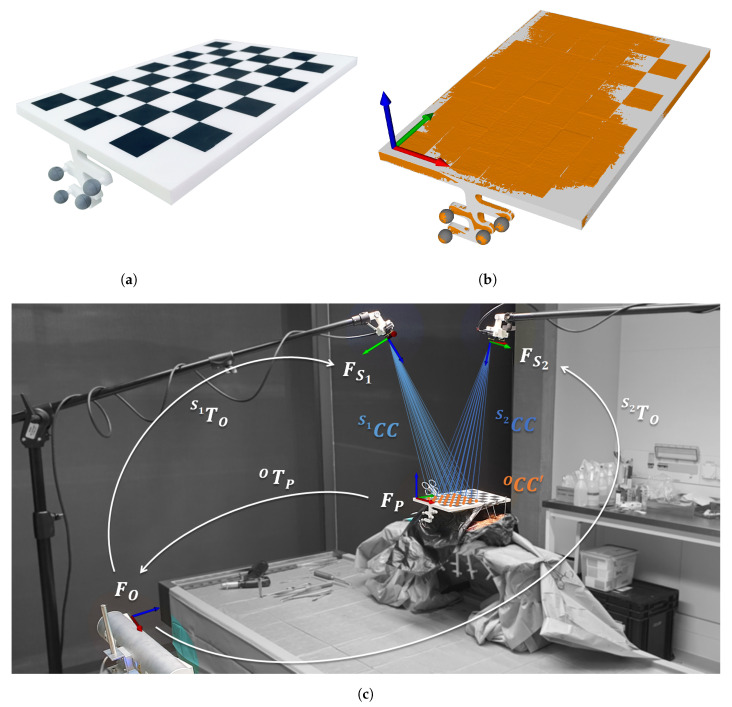
Extrinsic calibration materials and process. (**a**) 3D printed and painted calibration phantom with infrared-reflecting spheres mounted. (**b**) CT-extracted calibration phantom 3D model (orange), aligned re-calibrated marker geometry 3D model combined from generic sphere 3D models of true size (gray) and aligned CAD-exported calibration board 3D model (white) with its chessboard pattern origin w.r.t. which the four sphere centers (gray) were stored as the re-calibrated marker geometry. (**c**) Calibration phantom positioned on top of the anatomy such that optical tracking system (OTS; $F_{O}$) and RGB-D sensors ($F_{S_{1}},F_{S_{2}}$) can detect their respective part. 25 chessboard corners detected by either RGB-D sensor (${}^{S_{1}}CC,{}^{S_{2}}CC$) and their respective OTS counterparts (${}^{O}CC^{\prime }$), found by multiplying the ground truth 3D chessboard corners with ${}^{O}T_{P}$. Transformations ${}^{S_{1}}T_{O}$ and ${}^{S_{2}}T_{O}$ are found by Equation (2).

Estimating the rigid transformations between OTS and each RGB-D sensor (${}^{S_{i}}T_{O}$) requires an extrinsic calibration process. While the OTS relies on infrared emission and detection, each RGB-D sensor uses passive stereoscopy from two RGB images. Therefore, a calibration phantom incorporating both domains was designed and 3D printed (Figure 2a; Formiga P100 3D printer, EOS GmbH Electro Optical Systems, Krailling, Germany). The phantom combined a standard chessboard pattern of size 6 columns × 9 rows (square side length 30 mm) and snap fit mounts for four co-planar infrared-reflecting spheres forming a specific marker geometry. The mounts were placed on a branch orthogonal to the chessboard plane, facilitating simultaneous detection of the sphere markers by the OTS and the chessboard by the RGB-D sensors during the extrinsic calibration process. Since the material used for 3D printing was white polyamide 2200, black chessboard squares were printed as separate tiles and painted black manually. The tiles were then inserted into the foreseen notches on the board with a press fit.

Although the calibration phantom geometry and mount positions were known by design, 3D printing and manual sphere mounting could induce deviations from the CAD drawing. Hence, after mounting the sphere markers (Ø11.5 mm; ILUMARK GmbH, Feldkirchen b. München, Germany), the marker geometry was re-calibrated with the built-in functionality of the OTS that minimizes 3D marker registration error. To account for manufacturing inaccuracies, the phantom was then CT scanned (SOMATOM Edge Plus, Siemens Healthcare, Erlangen, Germany, slice thickness: 0.75 mm, in-plane resolution: 0.5 × 0.5 mm) to extract its 3D model using the global thresholding functionality of a commercial medical imaging software (Mimics Medical, Materialise NV, Leuven, Belgium). In an in-house developed preoperative planning software (CASPA, Balgrist University Hospital, Zurich, Switzerland), the re-calibrated marker geometry was generated from generic sphere 3D models of true size and combined to a single 3D model with a Boolean union operation [26]. This model was then aligned to the corresponding region, i.e., the spheres, within the CT-extracted phantom 3D model in an iterative closest point (ICP) fashion [27,28] (Figure 2b). Once this transformation is known, the re-calibrated marker geometry can be expressed in any coordinate frame. In order to facilitate extrinsic calibration calculations, the coordinate frame was chosen to be the origin of the chessboard pattern (Figure 2b), such that OTS and RGB-D sensors referred to the same real world location.

The extrinsic calibration process and the respective coordinate frames, transformations and point sets are illustrated in Figure 2c. Note that this is a one-time process, as long as the relative position of the RGB-D sensor and the OTS is maintained stationary. The calibration phantom was positioned on top of the anatomy such that the OTS could detect the sphere markers and the RGB-D sensors could detect the chessboard pattern. Along with the pose, the OTS provides a geometry registration error. If it was below 0.1 mm, the phantom pose for the OTS was stored as ${}^{O}T_{P}$. Otherwise, the phantom position was slightly varied until the error criteria was fulfilled. For each of the RGB-D sensors, the chessboard pattern was detected in both, the left and the right RGB camera frame using OpenCV’s (version 4.2) findChessboardCorners functionality [29]. Based on the intrinsic and extrinsic camera parameters of the RGB-D sensors, computed with a manufacturer-provided application, the inner 40 chessboard corners could be determined in 3D using OpenCV’s triangulatePoints functionality: ${}^{S_{1}}CC$ and ${}^{S_{2}}CC$. The corresponding points for the OTS are inferred by multiplying the ground truth 3D chessboard coordinates by ${}^{O}T_{P}$, yielding ${}^{O}CC^{\prime }$. Thereby, even a small error in ${}^{O}T_{P}$ propagates with each row of the chessboard. Preliminary experiments indicated the removal of the last three rows from all point sets to increase accuracy while maintaining robustness. The extrinsic calibration between OTS and each RGB-D sensor was then found with Horn’s absolute orientation method [30,31]:

(2) $${}^{S_{i}}T_{O}=\min\sum_{j=1}^{25}{∥R\times {}^{O}CC_{j}^{\prime }+T-{}^{S_{i}}CC_{j}∥}^{2},\;\;i\in \left\{1,2\right\}$$

where *R* and *T* are the rotational and translational components of ${}^{S_{i}}T_{O}$ and × denotes multiplication.

### 2.4. Cadaver Preparation

*Subjects:* Ten fresh-frozen human cadaveric spines were used for data collection. Two different types of exposure were investigated. Specimens 2–10 had history of spinal fusion performed within the scope of another research project. As a result, they had been freed from soft tissues such as the paravertebral muscles to expose the dorsal bony anatomy. Care had been taken not to damage the intraspinous ligament, the ligamentum flavum as well as the facet joint capsule. These specimens were defined as type “full exposure”. In specimen 1, all soft tissue structures were intact and a standard midline approach for instrumentation of pedicle screws from level TH12-S1 had been performed previous to our experiments (type “midline approach”). For all specimens, individual 3D models of each vertebra in its original condition were available. They had been extracted from a CT, acquired in the same way as for the calibration phantom (Section 2.3), using the global thresholding as well as the region growing functionality of Mimics Medical. They are referred to as *preoperative 3D bone models*.

**Figure 3:**
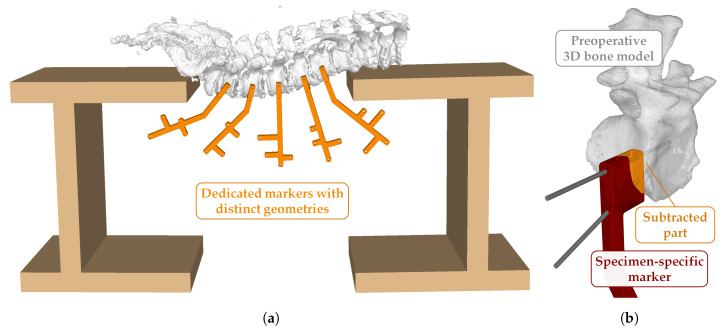
Setup for individual tracking of levels L1–L5. (**a**) Dedicated markers with distinct geometries (orange), designed and positioned such that they neither interfered with one another nor the C-shaped wooden jig. (**b**) Specimen-specific marker (red). The part for fixation was molded as a mirror model, such that it could fit to the vertebrae in an specific position and orientation. It was achieved by subtraction of the 3D vertebra model (white) from its corresponding marker 3D model (orange) using Boolean difference operation. Two slightly converging drill canals allowed for fixation through K-wires.

*Marker construct design:* Five dedicated markers were designed for individual tracking of levels L1–L5. The markers had four branches for distinct geometries and were shaped according to their position within the setup in a way that they neither interfered with one another nor the C-shaped wooden jig (Figure 3a). The part for fixation to the anatomy followed the key concept for registration in patient-specific instruments [32]. It was molded as a mirror model, such that it could fit to the vertebrae of interest in an specific position and orientation. This was achieved by subtraction of the vertebra from its corresponding marker 3D model using Boolean difference operation [26]. Two slightly converging drill canals at the top of each marker allowed for fixation to the vertebral bodies with K-wires according to the specimen-specific plan (Figure 3b). For each specimen, the *preoperative 3D bone models* were used in the CASPA software to create the specimen- and vertebra-specific marker constructs. The resulting 3D models were printed in the same way as the calibration phantom (Section 2.3). After manufacturing, the markers were equipped with infrared-reflecting spheres (Ø 6.4 mm; OptiTrack, NaturalPoint Inc., Corvallis, OR, USA).

*Anatomy dissection and marker attachment:* On each specimen, the left ventrolateral vertebral bodies were freed from the psoas muscle and the attachments of the diaphragm. The anterior longitudinal ligament was mobilized to the midline without damaging it. The marker constructs were placed at their planned position and fixated. The lower parts with the mounted marker constructs were wrapped into small plastic bags for protection from cadaveric tissue or fluid. The specimen was attached to the C-shaped jig with surgical pins. The C-shaped jigs were then rigidly locked to the table using G-clamps. After removal of the plastic bags, the marker geometries were re-calibrated, with the built-in functionality of the OTS that minimizes 3D marker registration error, thereafter the specimen was ready for data acquisition.

### 2.5. Data Acquisition

The data acquisition process for a single specimen consisted of three phases: recording the pedicle screw placement (i.e., surgical dataset), recording the frames required for Target Registration Error (TRE) analysis (i.e., TRE dataset) and acquiring a postoperative CT. The phases were always executed consecutively and are explained in the following. Throughout the process, the intrinsic and extrinsic camera parameters of the RGB-D sensors were re-computed regularly with a manufacturer-provided application.

During the recording, the OTS tracked the five marker geometries at its maximum frequency of ∼335 Hz (i.e., ${}^{O}T_{M_{j}}\left(t\right)$). The marker poses were stored with a system timestamp *t* captured directly after pose reception for each frame. Simultaneously, the RGB-D sensors captured the scene at a frequency of 30 Hz with a resolution of 1920 × 1080 pixels. The resulting data were stored in Stereolabs SVO files, containing entire video streams along with additional metadata such as system timestamps.

#### 2.5.1. Pedicle Screw Placement Recording (Surgical Dataset)

In each specimen, ten pedicle screws (M.U.S.T., Medacta SA, Castel San Pietro, Switzerland) were placed on vertebral levels L1–L5 (two screws per level, one in each pedicle) by a resident orhopedic surgeon. The insertion order of screws varied and is reported in the results section. For each screw, four surgical steps were conducted and recorded: (1) determining the correct entry point using anatomical landmarks and open bone cortex with a luer, (2) development of the screw canal with a surgical awl to a fully intraosseous trajectory, (3) verification of the integrity of bony canal with a K-wire and (4) placement of a measured screw implant in the previously established canal. For each pedicle, the following protocol was repeated until all screws were placed:

1. Reposition RGB-D sensors resembling a realistic surgical viewpoint;
2. Perform extrinsic calibration between OTS to RGB-D sensors, store ${}^{S_{1}}T_{O}$ and ${}^{S_{2}}T_{O}$;
3. For each surgical step:
  (a) Start recording;
  (b) Perform surgical step;
  (c) Stop recording.

For each specimen, 80 recordings (10 screws × 4 steps × 2 RGB-D sensors) were acquired.

#### 2.5.2. TRE Recording (TRE Dataset)

**Figure 4:**
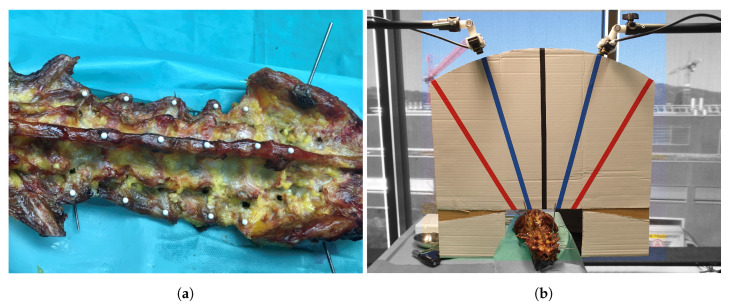
Setup for target registration error (TRE). (**a**) TRE were determined by push-pins, detectable in RGB-D frames and postoperative CT, inserted into both transverse processes and the spinous process of levels L1–L5. (**b**) Custom-made cardboard rule to set up standardized TRE viewpoints for RGB-D sensors at 0° (black), ±15° (blue) and ±30° (red).

For the later assessment of the TRE (Section 2.6), and after removal of all pedicle screws, the surgeon inserted a number of white push-pins (i.e., thumb tacks) into both transverse processes as well as the spinous process of levels L1–L5 (Figure 4a). A static scene was then captured from twelve standardized viewpoints, from which each RGB-D sensor covered six. First, the stereo baseline of the RGB-D sensors were aligned to the intersection of the axial and coronal planes, orthogonally facing the center of the surgical site at a distance of 600 mm. For the second viewpoints, the RGB-D sensors were inclined by +15° and −15°, respectively, and moved in superior/inferior direction such that they still faced the center of the surgical site at 600 mm distance. The third viewpoints were defined accordingly with inclinations of +30° and −30°. Viewpoints four to six followed the same protocol, but with the RGB-D sensors aligned to the intersection of the sagittal and coronal planes. All viewpoints were found using a custom-made cardboard template with angle markings (Figure 4b). After setting up each viewpoint, the OTS was calibrated to the RGB-D sensors and ${}^{S_{1}}T_{O}$ as well as ${}^{S_{2}}T_{O}$ (Figure 2c) were stored. Finally, a single frame was recorded.

#### 2.5.3. Postoperative CT

After recording the surgical dataset and the TRE dataset, the specimen was dismounted from the C-shaped wooden jig. A postoperative CT was acquired in supine position, while special care was taken to ensure that the marker constructs did not move relative to the specimen and the push-pins were not under heavy load in contact to the scanning table during this process. For specimens 1 and 2, the CT protocol was identical to the one described in Section 2.3. For the other specimens, the in-plane resolution was 0.6 mm.

### 2.6. Data Post-Processing

The postoperative CTs were processed using the Mimics Medical software. Table 1 provides an overview of the extracted data for each specimen and the functionality used.

**Figure 5:**
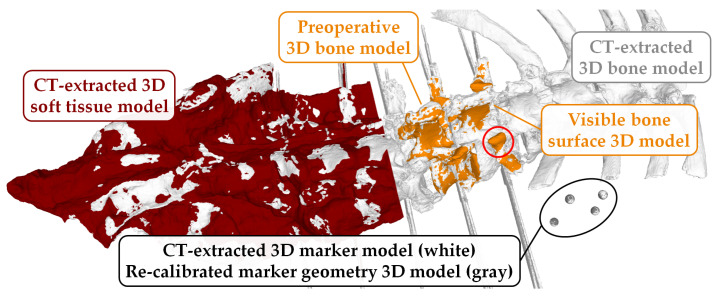
CT-extracted 3D marker model for L1 (white spheres) aligned to re-calibrated marker geometry 3D model combined from generic sphere 3D models of true size (gray); *preoperative 3D bone model* for L2 (orange) aligned to CT-extracted 3D bone model (white); CT-extracted 3D soft tissue model (red, cut off around L3 for visualization purposes) and visible bone surface 3D model for L1 (result when subtracting 3D soft tissue model (red) from *preoperative 3D bone model*). Red circle: area around the facet joint and mamillary process, which was present in the *preoperative 3D bone models*, but mostly absent in specimens 2–10 (history of spinal fusion performed within the scope of another experiment).

*Establishing the transformation chain*: A prerequisite for establishing the transformation chain (Equation (1)) is the calculation of the transformation between the specimens’ CT scan and the attached marker constructs (i.e., ${}^{M_{j}}T_{L_{j}}$). Although an approximation of this transformation could be achieved based on the marker constructs’ 3D design (Section 2.4), such approximations would include the rapid prototyping and attachment errors; therefore, we opted for measuring this transformation based on a postoperative CT of the specimen together with the attached marker construct. To this end, the four reflective spheres of each marker construct were extracted from the postoperative CT (Table 1, Figure 5), effectively resulting in an accurate measurement of ${}^{M_{j}}T_{L_{j}}$ (Figure 1). Furthermore, for every RGB-D frame in every recording, the OTS frame with the closest timestamp was found.

*TRE dataset preparation:* As indicated in Table 1, the push-pin head centers were localized in the postoperative CT by manually aligning the cursor in the axial, coronal and sagittal views: ${}^{C}PP$. The counterpart 3D coordinates of each push-pin was determined in each RGB-D sensor’s space. To do so, OpenCV’s [29] SimpleBlobDetector functionality was used to detect push-pin heads in the left and right image of each single-frame recording. Based on the intrinsic and extrinsic camera parameters of the RGB-D sensors, the 3D coordinates of the push-pin head centers were found through triangulation (triangulatePoints, OpenCV [29]) of the detected blobs: ${}^{S_{1}}PP$ and ${}^{S_{2}}PP$ to be used to calculate the TRE as explained in Section 2.7.

*Data cleanup:* As the postoperative CT contained K-wire artifacts from the attached markers and push-pins for TRE evaluation, the *preoperative 3D bone models* were employed as the reference for bony anatomy of levels L1–L5. Each *preoperative 3D bone model* was aligned individually to the CT-extracted 3D bone model (Table 1, Figure 5). Furthermore, despite the full exposure in nine out of ten specimens, parts of the bony anatomy were covered by residual soft tissue. Therefore, for accuracy evaluation, 3D models only describing the visible bone surface were created. From each aligned *preoperative 3D bone model* the 3D soft tissue model (Table 1, Figure 5) was subtracted using Boolean operations [26] in CASPA, resulting in the desired 3D model of visible bone surface (Figure 5).

### 2.7. Outcome Measures

Three outcome measures were defined to describe the accuracy of our setup and the resulting dataset: TRE, visible bone surface error (VBSE) and surgeon’s rating (SR).

**TRE**  The TRE was defined as the 3D Euclidean distance between the CT-extracted push-pin head centers ${}^{C}PP$ and their respective counterparts in each RGB-D sensor’s space: ${}^{S_{1}}PP$ and ${}^{S_{2}}PP$ (Section 2.6). As explained in Section 2.6, the 3D fiducial centroid coordinates in the RGB-D space were explicitly triangulated based on the stereo camera setup of the RGB-D sensor (based on intrinsic and extrinsic camera parameters) and we did not rely on the default stereo reconstruction algorithm of the sensor for these measurements. This was done so that the TRE measure only reflected on the errors associated with the data acquisition setup (e.g., extrinsic calibration) and not the inherent point cloud estimation of the sensor. The fiducial coordinates obtained from the CT measurement (${}^{C}PP$) were transformed to the space of either RGB-D sensor by ${}^{S_{i}}T_{L_{j}}\left(t\right)$ (Equation (1)). Note that points were transformed according to the levels they were inserted (i.e., index *j*) and the pairwise distances were calculated. For each cadaver, 180 datapoints (15 push-pin head centers × 6 viewpoints × 2 sensors) were assessed.
**VBSE**  In contrast to the TRE measure, the VBSE estimates the overall accuracy of our setup including the stereo reconstruction algorithm of the RGB-D sensor in use. The aforementioned visible bone surface 3D models of levels L1–L5 were transformed to the space of either RGB-D sensor using the estimated transformation ${}^{S_{i}}T_{L_{j}}\left(t\right)$ (Equation (1)), which itself is based on the OTS tracking data, and a depth map was rendered using the code of [33,34]. Ideally, the reconstructed depth map of either RGB-D sensor should be identical to their OTS-based, rendered counterparts. Therefore, any deviations between the two 3D representations can be attributed to inherent reconstruction errors of the RGB-D sensor and the errors within the data acquisition setup (assuming that the OTS measurement errors can be neglected). Furthermore, this error was influenced by the varying presence of screws in the RGB-D recordings and their absence in the postoperative CT as well as the absence of the area around the facet joints and mamillary processes in specimens 2–10 (history of spinal fusion performed within the scope of another cadaveric experiment) in the RGB-D recordings and their presence in the employed *preoperative 3D bone models* (Figure 5). All such phenomena result in domain mismatch; therefore, the VBSE was defined as the median absolute difference between all non-empty pixels of the rendered depth map and their corresponding pixels in the depth map reconstructed by the RGB-D sensors (except pixels, where no depth was reconstructed). Using the VBSE measure for RGB-D frames where the surgeon’s hand or the surgical tools were occluding the sensors view over the anatomy was not possible. This is due to the fact that the RGB-D stream could not be segmented to parts were exclusively points of the anatomy were present. Therefore, the VBSE measure was calculated for the first 10 frames (roughly 1/3 s) of each recording, where the surgeon’s hand were not present in the sensors’ field of view.
**SR**  The SR is a qualitative rating by the surgeon. A random sample of four recordings per specimen (10%) was selected for this purpose. For each recording, the first frame was selected (t=1). The aligned *preoperative 3D bone models* (Figure 5) of levels L1–L5 were transformed to the space of either RGB-D sensor by ${}^{S_{i}}T_{L_{j}}\left(1\right)$ (Equation (1)). Point clouds were extracted from the respective RGB-D frames. The surgeon then assessed the alignment of the bone models, i.e., the ground truth of our dataset, with the point cloud after the criteria:
  - Are the spinous processes in line?
  - Are the facet joint in line?
  - From a lateral view: are the vertebra at correct height?
  - Is the overall alignment correct?
  Each criteria received a score between 1 (worst) and 6 (best). The average of the four scores yielded the SR for one recording.

### 2.8. Depth Correction

Preliminary TRE analysis revealed small, systematic deviations between the transformed CT-extracted push-pin head centers ${}^{L_{j}}PP$ and their RGB-D counterparts ${}^{S_{1}}PP$ and ${}^{S_{2}}PP$ (Section 2.6) along the *z*-axis. This was attributed to the errors in the estimated intrinsic and extrinsic RGB-D camera parameters. Therefore, similar to the practice in RGB-D datasets reported in [24,25], a depth correction was deemed necessary. For each specimen and each sensor, the median *z*-coordinate deviation was calculated over all 90 datapoints (15 push-pins × 6 viewpoints). The focal lengths of each RGB-D sensor were then slightly adjusted (3.3 pixels on average) in the respective intrinsic camera parameters, which had been estimated by the manufacturer-provided application, such that the median deviation was as close to zero as possible.

## 3. Results

Table 2 provides an overview of all recordings. A total of 299,556 frames were recorded from 200 viewpoints (10 specimens × 10 screws × 2 RGB-D sensors). For the TRE, 1620 datapoints were recorded (9 specimens × 15 push-pins × 12 viewpoints). For VBSE, 720 recordings (9 specimens × 40 recordings × 2 RGB-D sensors) were evaluated. The SR based on 72 datapoints (9 specimens × 4 recordings × 2 RGB-D sensors).

**Figure 6:**
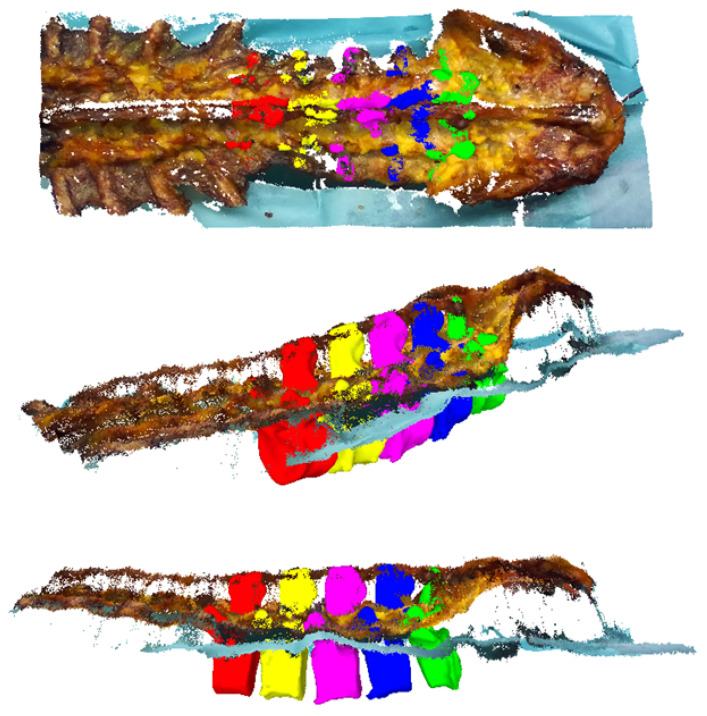
Point clouds and respective ground truth *preoperative 3D bone models* as generated for surgeon rating.

Results of the three outcome measures are summarized in Table 3. The overall mean TRE was 1.5±0.8 mm and the mean VBSE was 2.4±1.0 mm. The surgeon rated the ground truth alignment to the point clouds as 5.8±0.3 on average. Figure 6 shows exemplary point clouds and respective ground truth *preoperative 3D bone models* as generated for SR.

## 4. Discussion

Although standard CAOS systems have been shown to increase accuracy and patient safety in procedures such as pedicle screw placement, their rate of clinical adoption is low. Extensive registration of a preoperative plan to the intraoperative anatomy is seen as the major limitation associated with such technologies, thereby any technological advancement that can streamline the registration process and the underlying surgical guidance is welcomed in the community. Recent off-the-shelf RGB-D sensors can be the technology of choice for the next generation CAOS systems, given that they can provide accurate real-time 3D representation of the objects, therefore rendering the registration process obsolete. However, raw RGB-D representations are completely low-level (e.g., point cloud format) and cannot be used to address the surgical requirements. Current research in the field of RGB-D based surgical guidance still relies on conventional means of data registration (e.g., ICP registration of preoperative plan to the intraoperative points cloud), which is far from ideal in a real surgical setting. Given the emergence of AI methods that are capable of end-to-end 3D pose estimation and shape reconstruction, we see a notable potential for the employment of such methods for RGB-D-based surgical navigation. However, to the best of our knowledge, currently no medical dataset exists for training such algorithms that include corresponding RGB-D and anatomical pose data. In this study, we presented a data collection setup for pedicle screw insertion surgery captured by RGB-D sensors and a co-calibrated OTS, yielding 3D ground truth poses and shapes for spine levels L1–L5. Ten cadaveric specimens underwent surgery in an simulated surgical environment, resulting in a large dataset of (spinal RGB-D + pose) data, which we have made available together with this article.

In our setup, the multi-view RGB-D data stream was co-registered to real-time vertebral pose tracking data through implementing an extrinsic calibration procedure. This allowed access to continuous vertebral pose data even in cases where the RGB-D sensor’s filed of view was obscured (e.g., by the surgeon’s hand or surgical instruments). Based on the results of our quantitative accuracy assessment, a mean TRE of 1.5 mm of co-registration of RGB-D data and vertebral pose data was achieved, which is comparable to registration errors of state-of-the-art spine surgery navigation systems. The error for landmark-based registration initialization was 1.78 mm in [35] and 2.02 mm for landmark-based registration in [36]. However, various state-of-the-art navigation systems use surface-based registration algorithms. With this, after refinement, the error in [35] decreased to 0.70 mm and an error of 0.9 mm was reported in [37]. The most similar measure to surface-based registration errors in this study was the VBSE. With 2.4 mm, our setup’s VBSE was higher then the aforementioned errors reported in the literature. However, this comparison is only partly valid for two reasons. First, the VBSE reported in the herein study compares the co-registered RGB-D data to the CT-derived vertebral models using the OTS measurements; therefore, this error was estimated without conducting any further registration steps (such as the surface-based registration reported in [37]). Second, the error measure was estimated including all available dense 3D reconstruction of the anatomy, as opposed to [36]. In that study, anatomical registration was achieved based on dense 3D reconstruction from stereoscopic images captured through a surgical microscope; however, the reported reconstruction error (2.21 mm) was characterized based on 4 to 8 manually sampled points. Furthermore, in the currently existing public datasets of RGB-D images with labels of 3D shapes and poses, the median absolute deviation between ground truth and measured depth is commonly reported. In T-LESS [25], this error was comparable to ours with 2.5 mm for a structured light RGB-D sensor and 5.6 mm for a ToF sensor, respectively. The same sensors were employed in HomebrewedDB [24] and comparable errors of 2.56 mm (structured light) and 9.12 mm (ToF) were reported.

This study has limitations. First, the extrinsic calibration process used a single frame only and it was based on the intrinsic and extrinsic camera parameters found by a manufacturer-provided application. Despite re-calibration throughout the data acquisition, this may have influenced the collected data and/or resulted in inconsistencies. Furthermore, the large distances between anatomy and respective OTS markers are a likely error source. Additionally, the assumption about the the static transformation between the OTS system and the RGB-D sensors limits the use of the generated dataset to stationary RGB-D data. Furthermore, the estimated error measures where in scalar form and did not include direction-dependant components. Such measures are useful when preparing training datasets for designing context-aware and transparent AI methods.

As future work, a dynamic extrinsic calibration process will be developed that can provide the dynamic transformation between the OTS and the RGB-D sensors. This is useful when the RGB-D sensor cannot be maintained stationary in the operating room (e.g., worn by the surgeon in a form similar to a surgical headlight). Furthermore, in the future phases of this project, we are interested in providing RGB-D and pose data not only for the anatomy, but also for the surgical instruments (e.g., surgical drill, awl). Finally, using the generated data presented in this article, we will develop an AI algorithm that is capable of tracking the pose of each vertebrae based on intraoperative multi-view RGB-D data.

## 5. Conclusions

We have presented a multi-modal approach for acquisition of large surgical RGB-D video data along with ground truth 3D anatomy pose and shape labels. A respective RGB-D + Pose dataset of mock-up pedicle screw placement surgeries performed on ten cadaveric specimens in a realistic OR environment is made publicly available. Although the approach was tailored to a specific intervention and anatomy, it could be adapted to various procedures with reasonable effort. We have shown that such data can be collected with satisfactory accuracy and believe that once used by data-driven artificial intelligence methods, this dataset has the potential to build the basis for fast and reliable intraoperative registration.

## Figures and Tables

**Table 1:** Extracted data from postoperative CT for each specimen and used functionality.

Name	Quantity	Description	Functionality
3D marker model	5	Infrared-reflecting spheres for each marker attached to levels L1–L5	Global thresholding, region growing
Push-pin head centers	15	Push-pin head center coordinates	Manual cursor alignment in axial, coronal and sagittal views
3D bone model	1	Bony anatomy	Global thresholding, region growing
3D soft tissue model	1	Soft tissue including non-exposed parts of bony anatomy	Global thresholding, smooth mask, smart fill, erosion, manual artifact and push-pin removal, wrapping

**Table 2:** Overview of the recordings: type, screw order and total number of frames for each specimen. For each comma separated group, screws were inserted in ascending order of levels. Previously inserted screws were removed before moving on to the next group. If previously inserted screws are present in a subsequent group, these screws were not removed. If not indicated otherwise, screws were inserted bilaterally (left, then right) before moving on to the next level.

Specimen	Type	Screw Order	# of Frames
1	Midline approach	L1–L5	22,553
2	Full exposure	L1–L3, L4–L5	34,302
3	Full exposure	L1–L2, L3–L5 (LLL, RRR)	33,489
4	Full exposure	L1–L5	37,497
5	Full exposure	L1–L3, L4–L5	31,279
6	Full exposure	L3–L4, L4–L5, L1–L2	30,249
7	Full exposure	L4–L5, L1–L3	25,746
8	Full exposure	L2–L4, L4–L5, L1–L2	27,679
9	Full exposure	L1–L5	24,403
10	Full exposure	L4–L5, L1–L3	32,359

**Table 3:** Overview of the mean outcome measures target registration error (TRE), visible bone surface error (VBSE) and surgeon’s rating (SR) for each specimen.

Specimen	$\bar{\mathrm{TRE}}\pm \sigma$ [mm]	$\bar{\mathrm{VBSE}}\pm \sigma$ [mm]	$\bar{\mathrm{SR}}\pm \sigma$ [1–6]
1	N/A	N/A	N/A
2	1.9±1.1	3.9±1.4	5.6±0.4
3	1.7±0.8	2.6±0.6	5.8±0.3
4	1.6±0.8	2.5±0.5	5.8±0.2
5	1.6±0.8	1.8±0.3	5.9±0.2
6	1.3±0.6	1.6±0.3	6.0±0.1
7	1.3±0.6	1.8±0.3	5.8±0.3
8	1.1±0.5	1.8±0.4	5.9±0.2
9	1.4±0.7	2.6±0.7	5.8±0.3
10	1.6±0.7	2.7±0.9	5.4±0.4
$\bar{\mathrm{Overall}}\pm \sigma$	1.5±0.8	2.4±1.0	5.8±0.3

## Data Availability

The described dataset is available here: https://rocs.balgrist.ch/en/open-access/ (accessed on 26 August 2021).
